# Gene expression profiling of *Mycobacterium avium* subsp. *paratuberculosis* in simulated multi-stress conditions and within THP-1 cells reveals a new kind of interactive intramacrophage behaviour

**DOI:** 10.1186/1471-2180-12-87

**Published:** 2012-05-30

**Authors:** Andrea Cossu, Leonardo Antonio Sechi, Stefania Zanetti, Valentina Rosu

**Affiliations:** 1 Department of Biomedical Sciences, Division of Experimental and Clinical Microbiology, University of Sassari, Sassari, Italy; 2 Experimental Zooprophylactic Institute of Sardinia, Department of Nuoro, Nuoro, Italy

**Keywords:** *Mycobacterium avium* subsp. *paratuberculosis*, functional genomics, DNA-microarray, simulated intra-phagosomal multi-stress, macrophage infection

## Abstract

**Background:**

Recent studies have identified in *Mycobacterium avium* subsp. *paratuberculosis* (MAP), already known as a pathogen in ruminants, a potential zoonotic agent of some autoimmune diseases in humans. Therefore, considering the possible risk for public health, it is necessary a thorough understanding of MAP's gene expression during infection of human host as well as the identification of its immunogenic and/or virulence factors for the development of appropriate diagnostic and therapeutic tools.

**Results:**

In order to characterize MAP's transcriptome during macrophage infection, we analyzed for the first time the whole gene expression of a human derived strain of MAP in simulated intraphagosomal conditions and after intracellular infection of the human macrophage cell line THP-1 by using the DNA-microarray technology. Results showed that MAP shifts its transcriptome to an adaptive metabolism for an anoxic environment and nutrient starvation. It up-regulates several response factors to oxidative stress or intracellular conditions and allows, in terms of transcription, a passive surface peptidoglycan spoliation within the macrophage along with an intensification of the anabolic activity for lipidic membrane structures.

**Conclusions:**

These results indicate a possible interactive system between MAP and its host cell based on the internal mimicry unlike other intracellular pathogens, bringing new hypothesis in the virulence and pathogenicity of MAP and its importance in human health.

## Background

*Mycobacterium avium* subsp. *paratuberculosis* (MAP) is the causative agent of Johne's disease or Paratuberculosis, a chronic enteritis that mainly affects ruminants, causing a general debilitation of the infected organisms [1]. The disease is characterized by several phases that include, besides the initial phase of infection, a subclinical asymptomatic stage dominated by a Th1 type immune response, which usually is not able to eliminate the infection due to bacterial mechanisms of evasion [2], and then gradually replaced by a Th2 humoral immune response [3]. Since the humoral response is not able to fight against intracellular infection, the symptoms in the clinical phase becomes evident with the appearance of granulomatous lesions *in loco*, intestinal disorders and weight loss, culminating in the death of the infected animals [4].

Paratuberculosis seems to have many common features with the pathogenesis and the symptoms of Crohn's disease [5], a chronic inflammatory bowel disease that causes inflammation of the human gastrointestinal tract. As a matter of fact, although the bacterium has been recognized as a pathogen for poultry, ruminants and primates [6] extensive evidence such as the isolation of MAP in the intestinal tissue of Crohn's disease patients [7,8] and the presence of a humoral response to specific antigens of the bacterium in patients suffering from some autoimmune diseases [9] have suggested MAP as a potential human pathogen. MAP can survive for long periods under different environmental conditions [10] and is able to resist to several heat treatments conventionally used in the agricultural supply chain for transformation of various foodstuffs [11], moreover the bacterium is characterized by having a slow growth rate *in vitro*[8] and is capable to carry on a persistent infection with a slow course [12], that make it difficult to detect the infection with early diagnosis and microbiological cultural methods, respectively.

Most of the mechanisms underlying the development of disease caused by MAP have been explained following those based on diseases triggered by *Mycobacterium tuberculosis* (MTB) and *Mycobacterium avium* ssp. *avium*[13]. Mycobacteria infect mainly macrophage cells [14], for this reason they evolved to develop defense mechanisms to face the hostile environment they encounter within the phagosomal compartment. Consequently, the mycobacterial pathogens have developed a particular resistance to the common weapons of defense and destruction relied by phagocityc cells such as reactive nitrogen intermediates and oxygen radicals, the acidification of the phagosome and the release of antimicrobial peptides [15]. The main mechanism of defense implemented by the mycobacterium inside the macrophage is the inhibition of phagosomal acidification throught the prevention of phagosome-lysosome fusion, so that it may proliferate within it [16]. However, the molecular mechanism by which the mycobacteria are able to avoid the occurrence of phagolysosome maturation is still unknown. For this reason, many studies concerning the transcriptional regulation of macrophages infected by MAP have already been carried out [17,18] by using DNA-microarray technology that has become by now a useful tool also for the study of MAP gene expression under different environmental conditions [19] and during infection of bovine cell lines [20,21]. Additionally, the importance of MAP in terms of zoonotic relevance is recently gaining considerable attention especially in some autoimmune diseases where the bacterium could be involved [9,22]. In light of this, the characterization of MAP transcriptome during infection of human macrophage cell lines would be of great help in bridging a gap still present in the state of the art for this organism.

In order to characterize the transcriptional response of MAP under specific stress conditions, we analyzed by DNA-microarray the whole MAP transcriptome in acid-nitrosative multistress conditions as well as for the first time after intracellular infection of the human macrophage cell line THP-1.

Acid-nitrosative multi-stress is one of the most drastic antimicrobial stress operated *in vivo* by phagocytic cells against mycobacteria. By combining data from a simulated acid-nitrosative multi-stress in growth medium with those belonging to an *in vivo* intracellular stress, it could be possible to identify genes probably activated in a response to a radical stress and those induced by a more complex and articulated intracellular condition. The comparison of the two transcriptional repertoires may help understand the metabolic, regulatory and virulence patterns of this putative human pathogen. Results will allow the identification of possible key factors that may lead to the development of new diagnostic or therapeutic tools.

## Methods

### Bacterial cultures and growth media

*Mycobacterium avium* subsp. *paratuberculosis* (Linda strain) (ATCC 43015), originally isolated from a patient with Crohn's disease [23], was cultured in Middlebrook 7H9 medium (Sigma), 0.2% glycerol (Sigma), 0.05% Tween 80 (Sigma) supplemented with 10% v/v albumine dextrose catalase (ADC, Sigma) and 2 mg/L of Mycobactin J (MicJ) (Allied Monitors, Fayette, MO, USA) in 25 cm^2^ vented tissue culture flasks at 37°C.

### Acid-Nitrosative multi-stress

MAP's transcriptome in acid-nitrosative stress conditions were examined in 7H9-ADC medium. Early log-phase mycobacteria were exposed to the stress for 3 hours at 37°C. The acid-nitrosative stress was performed with a final concentration of 5 mM of sodium nitrite (NaNO_2_) (Sigma) in a buffered pH 5.3 broth supplemented with MicJ. After stress, cells were quickly harvested and resuspended in RNA later solution (Ambion) to preserve bacterial RNA. Bacterial pellets were then incubated overnight at 4°C and stored at −80°C until RNA extraction. Acid-nitrosative stress condition and relative control (untreated bacteria in 7H9-ADC-MicJ growth medium) were grown in triplicate and the entire process was repeated in a second experiment.

### Infection of THP-1 cells with MAP

THP-1 cells, a human monocyte cell line (ATTC TIB-202), were grown in T75 vented flasks (DB, Falcon) in RPMI-1640 medium (Invitrogen) supplemented with 10% heat-inactivated fetal bovine serum (Sigma) and antibiotic-antimycotic solution (1X) (Sigma) at 37°C under an atmosphere of 5% CO_2_. Cells were differentiated into macrophages with 50 ng/ml of phorbol 12-myristate 13-acetate (PMA) (Sigma) when they reached a concentration of 5x10^5^ cells/ml, and incubated for 24 h to allow differentiation. The next day adherence was confirmed by microscopy and monolayer was ready to be infected.

Infections were performed in T75 vented flasks containing monolayers with a confluence of approximately 1x10^5^ cells/cm^2^. Monolayers were washed 3 times with sterile PBS to remove antibiotics and then 25 ml of fresh medium were added to the monolayer before infection. Inocula for infection were prepared by centrifugation (5000 x *g*, 15 min) of 10 ml of MAP culture with a density of 8x10^8^ bacteria/ml. Bacterial pellet was resuspended in 10 ml of pre-warmed RPMI medium at 37°C and cells were declumped by 10 passages through a 21 gauge needle. Monolayers were infected by MAP with a multiplicity of infection (MOI) of 10:1 for 24 h at 37°C at 5% CO_2_. The next day, extracellular bacteria were killed by amikacin (Sigma) treatment (200 μg/ml) for 2 h at 37°C as already described [24,25]. Supernatant was removed and monolayer was washed with 3 x PBS rounds. By microscopic examination no extracellular bacteria were detected. Infected cells were selectively lysed by addiction of 10 ml of lysis buffer per monolayer (4 M guanidine thiocyanate, 0.5% Na N-lauryl sarcosine, 25 mM sodium citrate, and 0.1 M β-mercaptoethanol) without killing intracellular bacteria as previously described [24,25]. Flasks were shaked at 100 rpm for 15 min at room temperature (RT) and recovered lysate was thoroughly vortexed for 2 min before being passed five times through a 21 gauge needle to shear infected cells and reduce viscosity. One hundred milliliters of lysate belonging to ten T75 flasks were centrifuged at 5000 x *g* for 30 min at 14°C and pellet was resuspended in 1 ml of fresh lysis buffer. A final centrifugation at 10000 x g for 2 min was performed to harvest bacterial cells and pellet was then stored at −80°C until RNA extraction.

### RNA extraction

RNA was extracted by using the RiboPure-Bacteria Kit (Ambion) following the manufacturer's instructions with some modifications. Briefly, approximately 1x10^9^ mycobacterial cells were resuspended in 350 μl of RNAWIZ solution (Ambion) and transferred to a 0.5 ml skirted screw-capped microcentrifuge tube containing 300 μl of ice-cold Zirconia Beads. Tubes were immediately processed in the RiboLyser FP120-HY-230 RNA Lysing machine (Hybaid) for three cycles (30 s at speed 6.5) with cooling on ice for 1 min between pulses. Remaining steps were performed according to the manufacturer's instructions. RNA yield and purity was evaluated with the Nanodrop spectrophotometer (NanoDrop1000, Thermo Scientific) while RNA quality was examined by denaturing gel electrophoresis. All RNA samples were treated with Dnase I (Ambion) to remove trace amounts of genomic DNA.

### mRNA enrichment and linear amplification of mycobacterial RNA

The 16S and 23S ribosomal RNAs were removed from total RNA (tot-RNA) by using the MICROBExpress Bacterial mRNA Purification Kit (Ambion). Ten micrograms of input tot-RNA were used to get an average of 1–2 μg of output enriched mRNA. rRNAs removal was confirmed by denaturing gel electrophoresis. Enriched mycobacterial mRNA was then amplified by using the MessageAmp II Bacterial Kit (Ambion) with amino-allyl-UTP in order to incorporate modified uracyl nucleotides into amplified RNA (aRNA) during the IVT reaction to allow subsequently fluorescence dyes coupling.

### Labelling of aRNA

Fourty micrograms of aRNA were labelled with Alexa Fluor dyes 647 or 555 (Invitrogen) respectively for control samples and for experimental samples, following the manufacturer's protocol. Purification of coupled aRNA was performed by RNeasy purification system (Qiagen) and incorporation of dye was evaluated using Nanodrop. Before hybridization, coupled aRNA was fragmented using RNA fragmentation reagents (Ambion) following manufacturer's protocol.

### Microarray hybridizations

Microarray slides were purchased from Biodiscovery LLC (Ann Arbor, MI, USA). MAP K10 expression microarray contains one probe per gene for a total of 4337 probes covering 99.7% of all genes with 4 probe replicates per array in a 3 arrays format per slides for a total of 3x20K per slide. Each hybridization has been prepared following the Recommended Sample Preparation and Hybridization Protocols for Use with MYcroarrays (Biodiscovery LLC) with some modifications. Briefly, an hybridization solution of 220 μl (66 μl of 20X SSPE (3 M NaCl, 20 mM EDTA, 118.2 mM NaH_2_PO_4_, 81.8 mM Na_2_HPO_4_), formamide (10%), BSA (0.01 mg/ml), Tween-20 (0.01%), DTT (1 mM), manufacturer control oligos 1%, 10 μg of each target coupled-aRNA, RNAse free water until final volume) was prepared and pre-warmed at 56°C before hybridization. All hybridizations were carried out in a water bath at 55°C for 18 h in OneArray Sealed Hybridization Chambers (PhalanxBio Inc., Palo Alto, CA, USA) applicated to array slides following manufacturer's protocol. After incubation, microarrays were washed at RT with two rounds of SSPE 1X with Dithiothreitol (DTT) (0.1 mM) for 2 min, a 30 s final wash of SSPE 0.25 X with DTT (0.1 mM) and dried with spray air before been immediately scanned. All scans were carried out with an Axon 4200A scanner (Molecular Devices) at 5 μm resolution with full dynamic range of signal intensities at 1–65,000 in two-color mode (635 nm and 532 nm filters).

### Microarrays data analysis

Scanned images were obtained using the GenePix 6.0 software (Molecular devices). The signal intensity of each gene in both colors was calculated by the mean of median intensity of each replicate spot for each gene in the array giving an average for each gene extrapolated from 4 single spot signals. Median intensity values were corrected by background subtraction and negative corrected intensities were set to 10. Data were further normalized using the ratio-based setting for GenePix and gpr files belonging to hybridization signals analyzed by GenePix software were then loaded into the Multi Experiment viewer (MeV) from TM4 software suite for subsequent expression analysis. All values were log2 transformed for further analysis and a minimum 2-fold change in the ratio intensities with a p-value <0.05 was considered significant for a differential gene expression. *In silico* analysis to define each metabolic pattern was achieved using the Kyoto Encyclopedia of Genes and Genomes (KEGG) [26] for the identification of the metabolic pathway for each entry, Microbial Genome Database (MBGD) [27] for comparative analysis and InterPRO database [28] for the gene metabolic functions.

### Reverse transcription and real time PCR

Reverse transcription was carried out at 42°C, using 1 μg of RNA, 0.025 μg/μl random primers and the GoScript™ Reverse Transcriptase (Promega), in a final volume of 20 μl, following the manufacturer's instructions. PCR primers were designed with the Primer3 web software and verified for non-specific annealing with primer-blast. Control reactions, lacking reverse transcriptase, were performed for every RNA sample. Real time PCR reactions were accomplished using the iQ™5 Real-Time PCR Detection System (Bio-Rad), in a total volume of 25 μl, using 5 μl of diluted cDNA, 200 nM each of gene-specific primers and the GoTaq® qPCR Master Mix (Promega) with SYBR green assay. After 2 min at 95°C, the PCR program consisted in 40 cycles at 95°C for 15 s and 60°C for 1 min. Fluorescence was measured at the end of the annealing/extension step. Reactions were run in triplicate for each gene and the specificity of the PCR products was verified by gel electrophoresis and melting curve analysis. Results were normalized to the gene 16s rRNA as endogenous control with data belonging to a reference sample consisting of untreated bacteria in 7H9 medium. Expression values were calculated using the 2^-ΔΔCT^ method and for each condition, RNA from three independent cultures was utilized to determine the mean and standard deviation. Primer sequences used in this study and Real-Time qPCR analysis of selected genes are provided in Table 1 (Table 1) and Table 2 (Table 2) respectively.

**Table 1:** Primer sequences used in this study

**Gene ID**	**Primer name**	**Primer sequence (5'-3')**
***rRNA 16s***	MAP 16S FW	GCCGTAAACGGTGGGTACTA
	MAP 16S RV	TGCATGTCAAACCCAGGTAA
***MAP 3738c***	MAP 3738c FW	CCCACATTGGGATATGAAGC
	MAP 3738c RV	CTGAGGATCCTGGAGACGAG
***MAP 3522***	MAP 3522 FW	CTACCGGGAGCGTTATGTGT
	MAP 3522 RV	ACGATGGACGCCCAACTA
***MAP 1643***	MAP 1643 FW	TGGAGCGAGAACTACCACCT
	MAP 1643 RV	CACAGGTGGATCAACGTCAT
***MAP 0654***	MAP 0654 FW	GCAGGACTACACCATCGTCAT
	MAP 0654 RV	GTAGTCCTCCGTCGCCTTCT
***MAP 1407***	MAP 1407 FW	CGCTGTACACCGGAAAGATT
	MAP 1407 RV	CCGGTATTGGTAGACCATCG
***MAP 1317c***	MAP 1317c FW	CAGGTGGTATTCGCCTTCTC
	MAP 1317c RV	ATGAACCCGATACCAATCCA
***MAP 1535***	MAP 1535 FW	GTGTTCGTCTACGCGTTGCT
	MAP 1535 RV	ACCATGTAGAGGCGGTCCAC
***MAP 2055***	MAP 2055 FW	GAAATATCAATGGCCGCAAG
	MAP 2055 RV	AAGTTCAGTCGCAGGTGTCC

**Table 2:** Correlation of gene expression data (fold changes) obtained by DNA-microarray and real time qPCR

**MAP acid-nitrosative stress transcriptome**						
**Gene ID**	**Gene name**	**Gene Product**	**Microarray fold change**	**P-value**	**Real Time-qPCR fold change**	**SD**
***MAP 3738c***	-	Methyltransferase type 12 / Cyclopropane-fatty-acyl-phospholipid synthase	-2.78465771	0.00216317	-2.89367248	0.17
***MAP 3522***	*oxyS*	Transcriptional regulator, oxyS	4.02084912	0.00065264	2.66363166	0.60
***MAP 1643***	*aceAb*	Isocitrate lyase	7.02500864	0.00052984	4.30330061	0.07
**MAP THP-1 infection transcriptome**						
**Gene ID**	**Gene name**	**Gene Product**	**Microarray fold change**	**P-value**	**Real Time-qPCR fold change**	**SD**
***MAP 0654***	*phoT*	Phosphate transporter ATP-binding protein	-42.44433187	0.02392446	-16.81349291	0.91
***MAP 1407***	*-*	ADP-ribose pyrophosphatase	69.43061281	0.04255943	27.68837536	0.74
***MAP 1317c***	*-*	Acid-resistance membrane protein	4.39998925	0.00351578	2.90831542	2.42
***MAP 1535***	*pgsA2*	CDP-diacylglycerol--glycerol-3-phosphate 3-phosphatidyltransferase	6.40855813	0.00166329	2.51498937	6.99
***MAP 2055***	-	Cystathione beta-lyase	-9.04737958	0.00004972	-36.48386353	0.64

Selected MAP genes were validated for their expression profile by Real-Time qPCR to corroborate similar results in microarray data. Three selected genes are shown for the MAP acid-nitrosative stress transcriptome whereas five genes are shown for MAP THP-1 infection transcriptome. Gene ID: Gene identification code; SD: Standard deviation.

### Microarray data accession number

All transcriptional profile files have been submitted to the GEO database at NCBI [NCBI- GEO:GSE32243].

## Results

### Differential transcriptome of MAP under acid-nitrosative multi-stress

**Figure 1:**
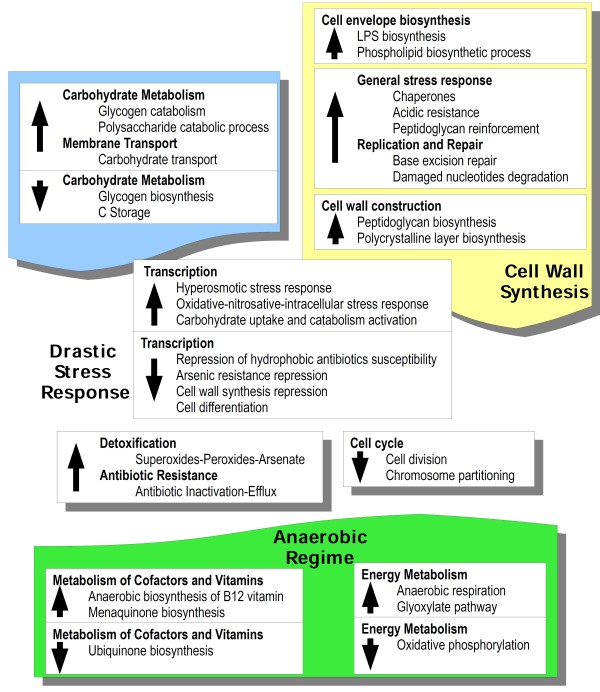
**Schematic diagram of MAP transcriptional response during acid-nitrosative multistress.** Differentially expressed genes during multi-stress were grouped based on the Kyoto Encyclopedia of Genes and Genomes (KEGG) classification and sorted by function. Up arrows indicate an up-regulation of genes to the related metabolism whereas down arrows indicate a down-regulation.

The whole transcriptome of MAP that has been highlighted during the acid-nitrosative stress (Figure 1) was defined by an up-regulation of 510 genes ( Additional file 1: Table S1) and a down-regulation of 478 genes ( Additional file 1: Table S2) for a total of 988 genes differentially expressed compared to the untreated strain. Transcriptional profile has been grouped into different types of metabolic patterns according to five functional class: intermediate metabolism, energy metabolism, cell wall & membrane, information metabolism and cell processes.

Within the intermediate metabolism category, the subgroup of amino acid metabolism is characterized by a significant up-regulation of the anabolic profile of several amino acids, such as branched-chain amino acids with subunits of ac*etolactate synthase 2* (*MAP4208, MAP3000c, MAP0649*), and specifically leucine (*leuA)* as well as an up-regulation of genes involved in the synthesis of aromatic amino acids (*aroK)* or specifically with entries for the synthesis of tryptophan (*trpE, trpB*) along with *tyrA* for the synthesis of tyrosine. Additional genes for the synthesis of amino acids are up-regulated, such as *ald* which is involved in the synthesis of alanine from pyruvate together with *dapA, dapB* and *dapE* in the synthesis of lysine, as well as the methionine's synthesis with *metA* and *methionine synthase (MAP3055c)*. Finally, in the same pattern there is an up-regulation of the synthesis of glutamine (*glnA3*) and some entries related to the synthesis of arginine (*argF, argH*).

### Multi-stress induces an increase in reserve polysaccharides degradation and in lipid anabolism

During acid-nitrosative stress, MAP up-regulates the catabolism of glycogen (*glgX, glgP*) along with two *glycoside hydrolase 15* (*MAP2215*, *MAP1384c*) which cleave the non-reducing terminal of dextrose-based polysaccharide complexes leading to D-glucose release. On the other hand, genes responsible for the synthesis of glycogen are repressed (*glgB, glgC*) as well as the synthesis of polyhydroxyalkanoic acids (PHAs) with the suppression of *poly-beta- hydroxybutyrate polymerase acid synthase* (*MAP1389*).

Regarding lipid metabolism, data show a notable shift towards up-regulation of genes involved in the biosynthesis of lipids rather than in the fatty acids degradation. As a matter of fact, genes for lipid biosynthesis are markedly up-regulated (*kas*, *fabG4, fabD2, desA2)* as well as *MaoC dehydratase (MAP3479c)*, *3-oxoacyl-carrier reductase* (*MAP3507), biotin carboxylase (MAP1701c*) and *diacylglycerol O-acyltransferase* (*MAP1156*) in the last step of triglycerides synthesis.

In line with this many genes for lipid catabolism are down-regulated. Among repressed entries are *AMP-dependent synthetase and ligase* (*MAP2400*, *MAP2747*, *MAP3659*) and *Acyl-CoA dehydrogenase* (*fadE1, fadE2*, *fadE15*, *fadE12*, *fadE3*, *fadE25, MAP2655, MAP2352*, *MAP0682*, *MAP2656*, *MAP2351*, *MAP1758c*, *MAP3238*) together with entries for *enoyl-CoA hydratase* (*echA7*, *echA21*, *echA6*, *echA12*) and the *patatin protein* (*MAP1011*), which is involved in the cleavage of fatty acids from membrane lipids, together with the *lipolytic enzyme G-D-S-L family* (*MAP1022c)* which is down-regulated as well.

Within the pattern of nucleotide metabolism it is interesting to note an up-regulation of the pyrimidine biosynthetic operon repressor (*pyrR*), for this reason MAP must make up for the loss of synthesis of pyrimidines through a bypass with *thyX,* required for the synthesis of dTMP, and *dcd* which is involved in the production of dUMP. An up-regulation can be observed also for *nrdI,* employed in the synthesis of deoxyribose and eventually in degrading damaged nucleotides with *NUDIX protein (MAP3088c)*.

With respect to the up-regulation pattern, where a repression of *pyr* operon was triggered, the *pyr* system which is involved in the classic synthesis of pyrimidines, coherently appears down-regulated (*pyrG, pyrF*).

As for the last subclass of intermediary metabolism, represented by the metabolism of vitamins and cofactors, an up-regulation of enzymes required for the synthesis of vitamin B12 was observed with *cbiX*, which participates in the anaerobic insertion of cobalt into the corrin ring, *cobyrinic acid a,c-diamide synthase* (*MAP3314c*), *cobW* and *cobT* required for the assembling of the cofactor's nucleotide loop in anaerobic metabolism. The synthesis of molybdopterin appears to be up-regulated (*mog, moeB*) as well as the synthesis of folate with entries such as *aminodeoxychorismate lyase* (*MAP1079*), *folE *and *folP*. The synthesis of menaquinone is up-regulated (*entC*, *menE, menC*) as well as the heme synthesis (*hemE, hemL*). Unlike from the up-regulation pattern, genes involved in the synthesis of FMN or FAD are repressed (*ribF*), in addition to the down-regulation of *lipA,* involved in the synthesis of lipoate and *ribokinase (MAP0876c*) in the synthesis of thiamine. Eventually, there is also a down-regulation of the synthesis of ubiquinone (*ubiX*) together with a suppression of the biotin synthesis (*bioB*) and coenzyme A synthesis (*coaA*) along with *5'-phosphate oxidase* (*MAP3177*, *MAP3028*, *MAP2630c*, *MAP0828*) related to the synthesis of vitamin B6.

### Stressor conditions induce in MAP an increase in anaerobic respiration and nitrate reduction

The energy metabolism of MAP during the acid-nitrosative stress includes the up-regulation of *eno*, which is involved in glycolysis, and some entries of the pyruvate dehydrogenase complex (*dlaT, pdhB, lpdA*). However, in this stress experiment, it seems that acetate originates also from the degradation of citrate with *citE* which is up-regulated. Furthermore some entries of Krebs cycle are also up-regulated (*gltA2icd2, sdhC*) together with some components of the electron transport chain such as *NAD(P)H quinone oxidoreductase* (*MAP0263c*), but with a different final electron acceptor than molecular oxygen with the up-regulation of *nirD* that reduces nitrite to ammonia and *periplasmic nitrate reductase* (*MAP4100c*) for nitrate as a final acceptor [29]. Alternative to Krebs cycle, but in parallel, MAP up-regulates components of the glyoxylate pathway with two entries such as *aceAb* and *isocitrate lyase* (*MAP0296c*).

Conversely, in the down-regulation pattern MAP represses oxidative phosphorylation by attenuating the expression of entries such as *atpC*, *nuoG*, *qcrB* and *fumarate reductase / succinate dehydrogenase* (*MAP0691c*) that together describe a repression of aerobic respiration with molecular oxygen as final electron acceptor during this stress.

The metabolism of transport in acid-nitrosative stress is represented by an up-regulation of genes involved in the uptake of cobalt such as *cobalt / nickel transport system permease protein* (*MAP3732c*) and *sulfonate / nitrate / taurine transport system permease protein* (*MAP0146MAP1809cMAP1109*) required for the transport of nitrate together with the transport of chloride with the up-regulation of *chloride channel protein* (*MAP3690*). During the stress there is an increase in iron storage with the up-regulation of *siderophore interacting FAD binding protein* (*MAP1864c*) although with two factors for iron uptake such *fecB* and *MAP3727*. Finally, a factor required for the uptake of carbohydrates such as *mannitol dehydrogenase* (*MAP0879c*) which belongs to the phosphotransferase system (PTS) [30] is up-regulated.

### Acid-nitrosative stress increases the expression of factors for the construction of lipid and glycan components of bacterial cell wall

Several genes involved in cell wall construction are up-regulated (*murA, murE, fbpC2*) along with *S-layer domain protein* (*MAP0951*) for the assembly of the surface polycrystalline layer of glycoproteins on the top of the lypoglican envelope [31], *D-alanyl-D-alanine carboxypeptidase* (*MAP0904*) and *ErfK / YbiS / YcfS / YnhG family protein* (*MAP3634*). It is important to note an up-regulation of the lipopolysaccharide (LPS) synthesis (*glf, rmlB2, rmlD*). Moreover, among up-regulated genes are *glycosyl transferase group 1* (*MAP1666c*), *exopolysaccharide biosynthesis tyrosine-protein kinase* (*MAP0952*) and *D,d-heptose 1,7-bisphosphate phosphatase protein* (*MAP3251*) required for the construction of the the inner core's precursor [32]. Finally, the biosynthesis of membrane phospholipids appears up-regulated in acid-nitrosative stress with entries such as *PA-phosphatase related protein* (*MAP1265*) together with *phosphatidylethanolamine N-methyltransferase* (*MAP3086c*), *phospholipid-binding protein* (*MAP1885c*), *phospholipid / glycerol acyltransferase* (*MAP3059c*), *diacylglycerol kinase* (*MAP3285c*) and *psd*.

It is worth noting that during the acid-nitrosative stress there is a repression of genes involved in the degradation of the cell wall such as *carbohydrate-binding protein* (*MAP0847*), *lytic transglycosylase* (*MAP4324c*), required for the degradation of murein in the cell wall recycling process during division and separation [33], *membrane-bound lytic murein transglycosylase* (*MAP2552*) and finally a couple of *transglycosylase domain protein* (*MAP0805c, MAP0974*) together with *mannan endo-1,4-beta-mannosidase* (*MAP1971*). In addition to these, a repression of cell division was inferred, since c*ell division FtsK / SpoIIIE* (*MAP4321c*) for cytokinetic ring assembly [34], *wag31* and *ATPase involved in chromosome partitioning* (*MAP3043c*) were down-regulated along with a *protein of unknown function DUF881* (*MAP0014*) involved in the division process.

Finally, there is a down-regulation of the synthesis of mycolic acids consistent with the repression of *inhA, mmaA4, kasB* and *methyltransferase type 12 / Cyclopropane-fatty-acyl- phospholipid synthase* (*MAP3738c*) in the synthesis of cyclopropane fatty acids.

### MAP triggers an oxidative stress-like response and suppresses the susceptibility to antibiotics during acid-nitrosative multi-stress

The subcategory of the information metabolism during acid-nitrosative stress is characterized by the up-regulation of *phoP* recognized as a positive regulator for the phosphate regulon as well as a virulence factor in MTB [35]. Several transcription factors are up-regulated during the stress such as *protein of unknown function YGGT* (*MAP1890c*) thought to be activated in response to hyperosmotic stress [36], *transcriptional regulator CRP / FNR family* (*MAP0082*) which responds to various stress stimuli such as oxidative stress and nitrosative stress [37]; interestingly, among up-regulated entries are also *sigE,* induced by oxidative stress or during infection of macrophages [38] and *oxyS* as regulator of oxidative stress response that mimics *oxyR*[39]. It is important to note the up-regulation of transcription factors for activating the uptake and catabolism of carbohydrates such as *transcriptional regulator, araC family* (*MAP1652cMAP0223c*) along with *furB,* a key protein in the control of intracellular iron concentration.

Within the down-regulated transcriptional profile, it is worth noting the suppression of *rsbU* which makes possible, through the activation of *rsbV*, the release of *sigB* factor sequestered by *rsbW*[40], moreover among repressed entries is *sigH* that is one of the activators of *sigB*. It is interesting to notice that also *sigA*, an important sigma factor recognised as differently expressed in other studies [41-43] is repressed, along with several *transcriptional regulator, merR family* (*MAP1541MAP1543hspR*), that can be traced to a general stress of starvation maybe due to a partial stationary phase condition, and several *transcriptional regulator, tetR family* (*MAP1477c, MAP3052c, MAP2394, MAP0969, MAP3891, MAP2023c, MAP1721c, MAP3689, MAP0179c, MAP2262, MAP4290, MAP2003c*) involved in the suppression of the susceptibility to hydrophobic antibiotics such as tetracycline [44]. During the stress there is also a down-regulation of *transcriptional regulator, arsR family protein* (*MAP0661c*) required for the suppression of resistance to arsenic compounds together with the repressor of the cell wall synthesis *cell wall envelope-related protein transcriptional attenuator* (*MAP3565*). Finally, it is worth noting the repression of *whiB4*, which is useful for differentiation and cell division.

The last subgroup of the information metabolism is the signal transduction within which, during acid-nitrosative stress, transduction through kinases is up-regulated with *sensor signal transduction histidine kinase* (*MAP1101*), *pknGpknL*, together with *prrB* which is involved in the adaptation to a new environment or to intracellular growth [38].

MAP's metabolism of detoxification reveals an up-regulation of detoxification enzymes such as *sodC*, which is responsible for the degradation of superoxides, together with *katG* and *bpoC* for peroxides elimination, as well as *arsC* and *arsb2* for detoxification from arsenic acid or heavy metals [45]. It is important to note the up-regulation of the resistance to multiple antibiotics with several entries such as *aminoglycoside phosphotransferase* (*MAP2082MAP3197MAP0267c*), *antibiotic transport system permease protein* (*MAP3532c*) and *prolyl 4- hydroxylase, alpha subunit* (*MAP1976*) in the hydroxylation-mediated inactivation.

Regarding the subgroup of antigenicity and virulence, it is worth noting the up-regulation of *PE-PGRS family protein* (*MAP4144*) and several PPE proteins (*MAP0123MAP1516MAP1519MAP2595MAP3185MAP1003c*) thought to be responsible for the antigenic diversity [46]. Furthermore, several virulence factors required for cell invasion or escape are up-regulated such as *hemolysin* (*MAP1551c*) and *mce* (*MAP1857MAP0767cMAP3609*) together with a couple of *cutinase* (*MAP4237cMAP3495c*) perhaps involved in the destruction of the host cell membrane lipids [47].

On the other hand, data show the repression of several immunogenic factors (*mpt6, esxD, snm4, lprG*), all virulence factors but not necessarily immunogenic, suggesting a change in the antigenic profile of the bacterium, not due to a repression of the antigenic diversity, but to an alternative antigenic profile.

The response to acid-nitrosative stress is characterized by the up-regulation of many stress chaperonins (*DnaJHsp20GroESGroEL*) for the protein folding along with resistance factors such as *acid resistance membrane protein* (*MAP1317c*) for resistance to acids and three entries of *acyltransferase 3* (*MAP3276cMAP3514MAP1271c*) required for peptidoglycan O-acylation in order to increase its resistance [48]. There is also an up-regulation in the response to DNA damage with the activation of a not-SOS dependent repair system with *enduvrA* and *xthA* for the removal of damaged nucleotides [49], *uracil-DNA glycosylase* (*MAP3256c*) and *formamidopyrimidine-DNA glycosylase* (*MAP0889*) specific for oxidized purines [50]. Lastly, MAP's transcriptome under acid-nitrosative stress shows the repression of few general chaperonins, probably due to stationary phase starvation, such as *GroEL2* and *uspA* identified in "stress endurance" response not due to acute stress [51], as well as the down-regulation of *activator of Hsp90 protein family* (*MAP1640c*) and *htrA*, a heat shock protein together with *proW* for osmotic shock.

### Transcriptome of MAP during the infection of THP-1 human macrophages

The transcriptional pattern of MAP after *in vitro* infection of the macrophage cell line THP-1 showed a combination of metabolisms (2) defined by the expression of a total of 455 genes, 171 of which are up-regulated ( Additional file 1: Table S3) and 284 are down-regulated ( Additional file 1: Table S4).

**Figure 2:**
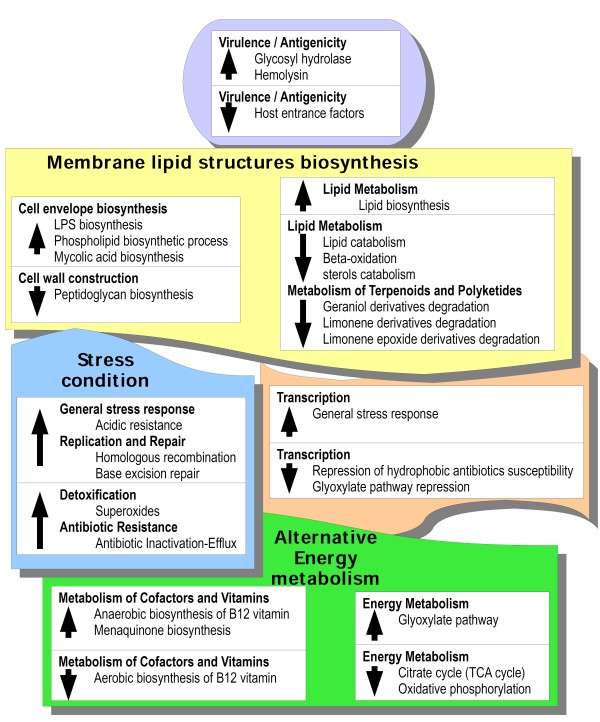
**Schematic diagram of MAP transcriptional response during THP-1 infection.** Differentially expressed genes during cellular infection were grouped based on the Kyoto Encyclopedia of Genes and Genomes (KEGG) classification and sorted by function. Up arrows indicate an up-regulation of genes to the related metabolism whereas down arrows indicate a down-regulation.

### Within macrophage MAP up-regulates amino acid catabolism, down-regulates amino acid anabolism and inhibits lipid degradation

It is interesting to notice that within the up-regulated framework there is an increased expression of genes involved in the degradation of asparagine (*ansA*), glutamate with *NAD- glutamate dehydrogenase* (*MAP2294c*) and phenylalanine with *mphA* and *fumarylacetoacetate hydrolase protein* (*MAP0881*). Moreover, it is important to note that the catabolism of cysteine is up-regulated with *cysteine desulfurase* (*MAP1190*), which is involved in the removal of sulfur to yield alanine, an important gene in the synthesis of S-based cofactors [52].

Differently, according to the down-regulated pattern, there is a clear shift towards the amino acid anabolism. Therefore, the synthesis of histidine is down-regulated with three entries such as *hisI*, *hisD* and *histidinol-phosphate aminotransferase* (*MAP4211).* Among down-regulated entries are also those required for the synthesis of methionine with four repressed genes such as *metC*, *metH, homocysteine methyltransferase* (*MAP2279*) and lastly *cystathione beta-lyase* (*MAP2055*). The synthesis of threonine seems down-regulated (*thrC*) together with the synthesis of glutamine (*glnA2*) and lysine with *dihydrodipicolinate reductase protein* (*MAP2013c, MAP3619*).

The metabolism of carbohydrates shows during THP-1 infection an up-regulation of *lpqI* which participates in the hydrolysis of beta-linkages in polysaccharides and the consequently release of free glucose.

The down-regulated profile shows rather the opposite process to the degradation of polysaccharides, although with formation of alpha-linkages, with *glgC* involved in the synthesis of glycogen.

The lipid metabolism is characterized by a slight up-regulation of the synthesis of fatty acids with *fabG2* and *MaoC domain protein dehydratase* (*MAP3479c*).

On the other hand during the THP-1 infection, MAP's degradation of lipids is heavily down-regulated with the repression of *fadD13*, *fadE6* and *acyl-CoA dehydrogenase* (*MAP3238*), as well as three entries for *enoyl-CoA hydratase* (*echA9*, *echA19*, *echA16*) and *fadA6*. Lastly, a gene involved in the degradation of sterols, *steroid delta-5-3-ketosteroid isomerase* (*MAP1773c*), is down-regulated.

### Intramacrophage environment brings MAP to employ mechanisms for energy production and cofactors biosynthesis through anaerobic pathways

As far as the metabolism of cofactors and vitamins is concerned, among up-regulated genes are those specific for the synthesis of folate such as *aminodeoxychorismate lyase protein* (*MAP1079*) and *dfrA* along with genes responsible for the synthesis of porphyrins (*hemE, hemZ*) for heme production. In addition, there is an increase in the synthesis of B12 cofactor through anaerobic process (*cobT*) together with the up-regulation of the synthesis of biotin (*bioF*) and the biosynthesis of menaquinone (*menB)*.

In opposite to the up-regulation profile, the synthesis of B12 cofactor under aerobic conditions is down-regulated with *cobN* required for the aerobic synthesis of its corrin ring, along with the the synthesis of coenzyme A with *coaA* and *dephospho-CoA kinase* (*MAP1326*). During THP-1 infection MAP up-regulates *acn* that is used both in tricarboxylic acid (TCA) cycle and in glyoxylate pathway. In addition there is also an up-regulation of the pentose phosphate pathway with *glucose-6-phosphate 1-dehydrogenase* (*MAP1687*).

On the other hand, among down-regulated genes are entries for TCA cycle (*gltA1, mdh*), as well as several entries for oxidative phosphorylation such as *nuoG, ndh* and *NAD(P)H quinone oxidoreductase (MAP0245c)* and ATP synthesis using molecular oxygen as final electron acceptor such as *ATP synthase I* (*MAP2458c*) together with *atpE*.

### Intracellular MAP increases the expression of factors related to polypeptides translocation and production of metal chelators

As far as the metabolism of transport is concerned, it is important to note an increase in genes involved in protein translocation with the up-regulation of entries such as *secG* and a couple of *peptide / nickel transport system permease protein* (*MAP1087MAP1088*) along with an up-regulation of factors concerning the transport of chloride such as *chloride channel protein* (*MAP3690*) and the “low-affinity” uptake of phosphate (*pitA*) [53] as well as *sulfonate / nitrate / taurine transport system permease protein* (*MAP1109*) involved in the nitrate transport. Finally, it is worth noting how *sugB*, which is responsible for sugar transport and uptake, is up-regulated together with *entB* required for capturing iron from host cell's iron chelator compounds [54].

On the other hand, the down-regulated expression profile shows a repression of the “forced” system of phosphate uptake (*phoH, phoT*, *pstA1_1, pstA1_2)* thus showing the repression both in the activation of the *pho* system and in the induction of the *pst* system. It is interesting to notice that the down-regulated pattern is also dominated by the repression of the uptake of cationic metals such as nickel (*nicT*) and molybdenum (*modC, modD*) and the transport of lipids which is suppressed with *mmpL11* and *mmpl protein* (*MAP2233*).

### Within macrophage MAP up-regulates genes for membrane lipids but not in peptidoglycan biosynthesis

The cell wall and membrane metabolism of MAP during the THP-1 infection is characterized by the up-regulation of genes involved in the synthesis of membrane lipid structures such as LPS with *D,d-heptose-1,7-bisphosphate phosphatase protein* (*MAP3251*) as well as entries required for the synthesis of phospholipids such as *phospholipid / glycerol acyltransferase* (*MAP1160c*), *1-acyl-sn-glycerol-3-phosphate acyltransferase* (*MAP1920c*), *hemolysin* (*MAP3059c*), *pgsA2* and *pgsA3*. Finally, there is also an up-regulation in the production of mycolic acids with *fbpC2* that is necessary for the biogenesis of the cord factor.

The down-regulated expression pattern is mainly featured by the suppression of the synthesis of peptidoglycan with genes such as *gmdA*, *murE, murG, murX* and *bifunctional phosphoglucose / phosphomannose isomerase* (*MAP3368c*). Along with the down-regulation of *maf-like protein* (*MAP3401*) responsible for the inhibition of the partitioning septum, thus suggesting a possible increase in cell division.

### Intracellular MAP increases the expression of genes involved in antibiotics resistance and radical agents as well as factors for cellular evasion, but not for invasion

Information metabolism is characterized by the up-regulation of genes concerning the regulation of sugar metabolism such as *transcriptional regulator, araC family* (*MAP3758c*, *MAP1652c*) and *transcriptional regulator, gntR family* (*MAP3599c*) that regulate the biosynthesis of sugars. The last up-regulated entry is *transcriptional regulator, merR family* (*MAP3267c*) which is important for the response to oxidative stress and antibiotics.

Among the down-regulated genes are two sigma factors such as *SigI* which is activated in response to general stress and *SigJ*, required for the regulation of expression in stationary phase cultures [55]. The susceptibility to lipophilic antibiotics is repressed since four genes coding for *transcriptional regulator, tetR family* (*MAP3052cMAP0155MAP2262MAP0335*) are down-regulated along with the repression of the glyoxylate path with *transcriptional regulator, iclR family* (*MAP1446c*).

With respect to the detoxification metabolism during macrophage infection, MAP up-regulates *sodC* in order to dismutate superoxides, and increases its antibiotic resistance by up-regulating genes such as *aminoglycoside phosphotransferase* (*MAP3197*), *prolyl 4-hydroxylase, alpha subunit* (*MAP1976*) and *antibiotic transport system permease* (*MAP3532c*) for their efflux.

Virulence and antigenicity of MAP during infection of THP-1 are dominated by the up-regulation of *mpt64*, *tlyA*, *peptidase M22 glycoprotease* (*MAP4261*), and *family PE-PGRS protein* (*MAP4144*).

The *hbha* gene for host cell adhesion as well as *mce1C* for the invasion of mammalian host cells are down-regulated, thus limiting the invasive feature of MAP during intramacrophage infection. Lastly, there is a down-regulation of components belonging to antigenic variability such as four *PPE family protein* (*MAP0966c*, *MAP2927*, *MAP1515*, *MAP3737*) that are repressed.

The stress metabolism shows an up-regulation of *acid-resistance membrane protein* (*MAP1317c*) specific for resistance to acidic environment, *uspA* (*MAP1754c*) and two entries for the repair of damaged DNA such as *recR* and *end*.

On the other hand, within this metabolism two entries such as *Hsp20* and *dnaJ* are repressed along with *domain-containing protein PitT* (*MAP2680c*, *MAP2027c*) required for MAP's survival under nutritional stress.

### Comparison of acid-nitrosative multi-stress and THP-1 infection MAP's transcriptomes

**Figure 3:**
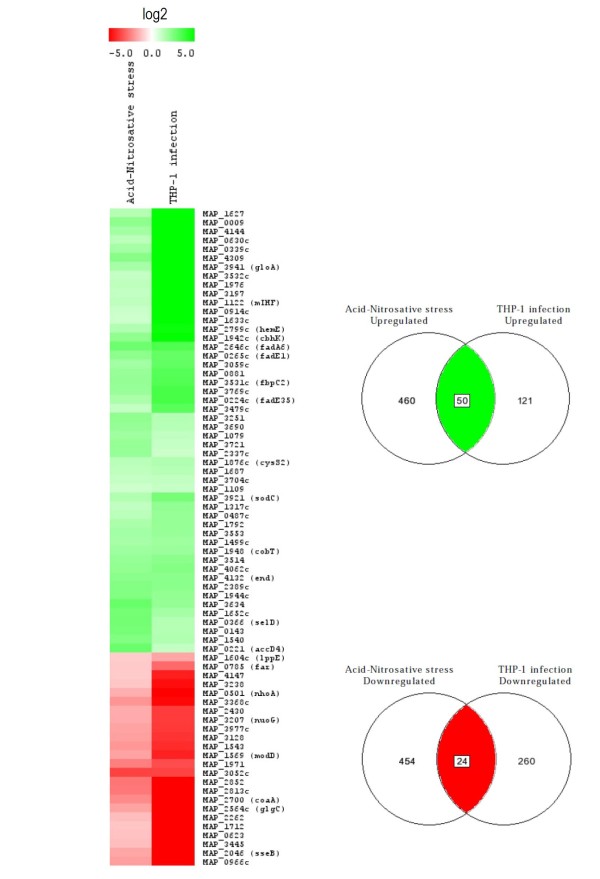
**Functional clustering of common regulated MAP genes under acid-nitrosative multi-stress and THP-1 infection.** Expression ratios were log2-transformed, and displayed according to the color code at the top of the figure. Venn diagrams showing the number of overlapping and unique genes modulated more than 2.0-fold under the two experimental conditions are on the right of each colored macrocluster. The number of induced or repressed overlapping genes is indicated in the green ellipse or red ellipse, respectively.

MAP's transcriptome resulting from the acid-nitrosative stress is more complex and rich (n = 988) than the detectable transcriptome during infection of the macrophage line THP-1 (n = 455). Between the two transcriptomes it is possible to find analogies of up-regulation or down-regulation for several entries since 50 and 24 genes are commonly up-regulated and down-regulated, respectively (Figure 3). Homologies can be found in the intermediate metabolism, where there is a repression of the synthesis of glycogen both in the acid- nitrosative stress (*glgBglgC*) and in the cellular infection (*glgC*), thus highlighting a limitation in extracellular sources of carbohydrates. In the lipid metabolism both transcriptional profiles underline an up-regulation trend towards the lipid synthesis (*MAP3479c*) together with a repression of lipid degradation (*MAP3238*), in broad agreement with other studies where lipid synthesis was already observed as up-regulated in experiments of multiple-stress in MTB [56] since they may serve as nutrient storage.

The down-regulation of pyrimidine synthesis is a common repressed metabolism between the acid-nitrosative stress and the infection especially in the first where the synthesis is repressed by the *pyrR* regulator resulting in a down-regulation of *pyr* genes, perfectly correlated with the same mechanism of genic regulation occurred in previous experiments inherent MTB's response to inhibitors of translation [19] in which it was shown that the translational inhibition induced the bacterium to trigger a response that included both the repression of *de novo* nucleotides synthesis and the increase of the synthesis of ribosomes.

Finally, the situation appears very complex in the common metabolism of synthesis of vitamins and cofactors in which the up-regulation of folate synthesis occurs in both transcriptional profiles with the same entry *aminodeoxychorismate lyase protein* (*MAP1079*) as well as the synthesis of vitamin B12 (*cobT*) and the synthesis of porphyrins (*hemE*). In this case, the up-regulation of porphyrins synthesis may be due to the situation of starvation that requires MAP to shift its energy metabolism from an aerobic condition to an anaerobic state using enzymes that cooperate with ferredoxines in the transfer of electrons in redox reactions as like as a metabolism pattern already identified in previous studies with the induction of slow growth and hypoxic cultures of *Mycobacterium smegmatis* (MSMEG) [57].

Further evidences about the switch of energy metabolism from aerobic pathway to anaerobic conditions are represented by the common up-regulation of the synthesis of menaquinone in both experiments, respectively with *menA* and *menB* in acid-nitrosative stress and in the cellular infection, since it could be an essential factor for the survival of non-replicating mycobacteria [58], thus corroborating the decrease of cell multiplication given by the down-regulation of functional genes for cell division. The only homology in the down-regulation profile of metabolism of cofactors is the repression of *coaA,* probably in line with the down-regulation of lipid degradation.

As far as the energy metabolism is concerned, both transcriptomes are characterized by the up-regulation of the glyoxylate pathway in particular in the acid-nitrosative stress with *aceAb*, which was identified in many works as a factor expressed by mycobacteria to survive inside the macrophage and in other infection models as well as during growth with lipids as the sole sources of carbon [59]. Nevertheless, the up-regulation of genes involved in the synthesis of lipids, especially in the construction of lipid membrane structures, is in contrast with previous works reporting that inside the macrophage mycobacteria, such as MTB, shifted their energy metabolism to the use of fatty acids in beta-oxidation [24].

However, the regime of anaerobic respiration is further confirmed by the down-regulation of oxidative phosphorylation both for subunits of *NADH dehydrogenase* and for other complexes involved in electron transport chain together with *F0F1 ATPase* subunits as already observed in experiments with MTB under nutrient starvation [60], oxidative agents [61] and in infection of macrophages [62] in addition to the common down-regulation of *nuoG,* which was identified in MTB as an antiapoptotic factor for macrophages [63].

In the complex metabolism of cell wall and membrane, both transcriptomes show a common up-regulation of the synthesis of LPS (*MAP3251*) and membrane phospholipids (*MAP3059c*) while in the cell processing metabolism, a common up-regulation of resistance factors to multiple antibiotics (*MAP3197MAP1976MAP3532c*), together with a common down-regulation of some *tetR* factors (*MAP3052cMAP2262*) involved in the suppression of the resistance to lipophilic antibiotics, is consistently present as similarly seen in MTB with multiple stress experiments [56]. Additionally, the detoxification metabolism underlines a common degradation pathway for reactive oxygen species with *sodC* which was also found to be significantly expressed in MTB during oxidative stress [61] together with the up-regulation of *acid-resistance membrane protein* (*MAP1317c*) in order to cope with the acidic environment, and *end* required for the repair of DNA damage, previously identified in MTB after treatment with antibacterial agents [64]. Finally, MAP's virulence exhibits a common up-regulation of the *PE-PGRS family protein* (*MAP4144*) in both transcriptomes which might be a common response to the antigenic diversity profile.

## Discussion

Most of the works present in the literature concerning studies on whole functional genomics in *in vitro* mycobacterial infection of mammalian cell lines have focused on the transcriptional framework of the infected cell rather than the transcriptome belonging to the infecting bacteria [17,18,65]. This is due to the fact that obtaining sufficient amount of RNA from mycobacteria in order to perform microarray hybridization experiments is difficult [21]. Furthermore, among the few studies concerning the transcriptome of MAP, no one has been focused on the MAP-human system, but only on the definition of the MAP transcriptome in bovine or murine cells [20,21,66].

Recent findings suggesting the putative role of MAP in the development of intestinal diseases in humans such as Crohn's disease [7,67,68] or immune system disorders such as type I diabetes [9,22], channel new research lines in the study of the bacterium's transcriptome during the infection of the potential human host.

For this reason this work has focused on the transcriptional profile of MAP in two types of environmental conditions. The first one was the simulation of the intraphagosomal environment by inducing a multiple stress system made by both the acid and the nitric components defining thus an acid-nitrosative environment with protonic and radicalic stressors, since the addition of nitrite to a growth medium at low pH, would have produced various anionic species of nitrogen oxides together with NO [69]. Consequently, the experiment conducted in the acid-nitrosative stress would have served to highlight the transcriptional regulation of the bacterium in growth conditions reproduced in the standard growth medium with the simulation of the macrophage internalization probably encountered during *in vivo* infection. On the other hand, the second experimental approach has seen the preparation of the infection system MAP-macrophage using the human macrophage/monocyte cell line THP-1 as host. By employing a simple and efficient protocol for the isolation of intracellular mycobacteria from infected cells [25] it was possible to get a good starting amount of bacteria through the specific lysis of infected eukaryotic cells, surprisingly resulting in a very viable bacterial pellet (data not shown), sufficient for downstream experiments starting from the extraction of bacterial RNA.

As far as the experimental transcriptomes are concerned, it could be noticed that under nitrosative stress as well as in macrophage infection MAP shifts its aerobic metabolism to a set of systems related to an energy metabolism based on the anaerobism, enabling nitrate respiration to generate ATP [70], unlike mechanisms such as the oxidation of molecular hydrogen with the hydrogenase complex [57]. This shift towards the nitrogen compound may be due in the case of multiple stress to the prevalence of nitrogen species in the culture medium ensuring that the bacterium utilizes the condition of excessive nitrate to its advantage, even though in a condition of starvation, using the nitrogen compound as an electron acceptor. Moreover, in the second case regarding the persistence of MAP in macrophages, since the phagosome is known to be an anoxic environment [71], in lack of molecular oxygen, the bacterium exploits oxidized nitrogen species in order to have an efficient anaerobic respiration. Common up-regulation of genes required for the synthesis of menaquinone in both experimental conditions along with the down-regulation of genes peculiar of the aerobic respiration, such as those for oxidative phosphorylation and synthesis of ubiquinone, corroborates this hypothesis since members of the menaquinone synthesis pathway have been found up-regulated four times in experiments of induced hypoxia with MSMEG [57]. This would confirm the belief that, during infection, the macrophage environment is dominated by a general condition of hypoxia as already demonstrated in MTB [72], and together with the here described down-regulation of MAP's TCA cycle would reflect a general slowing down of metabolism already found in MTB under induced conditions of nutrient starvation [60].

The perception of stress conditions in both experiments is emphasized by the up-regulation of several stress factors such as chaperonins and specific transcription factors among which it is worth to mention the *ad hoc* sigma factor *sigE* which is activated intracellularly or during oxidative stress [38]. It is important to note the up-regulation of *oxyS* required for the response to general oxidative stress and *sodC* in the acid-nitrosative stress, along with the response for the resistance to acids (*MAP1317c*). Of particular interest in THP-1 infection is the down-regulation in MAP transcriptome of the repressor of the glyoxylate cycle with the concomitant up-regulation of this pathway, which was identified as a characteristic feature of the persistence of mycobacteria inside the macrophage [73], along with the down-regulation of genes involved in the synthesis of glycogen and pyrimidines, commonly down-regulated in both experiments. Ultimately, this set of regulated genes pertaining to this part of the transcriptional pattern shows, how in line with several works [20,74], the bacterium expresses a specific defense against toxic compounds and an adequate response to the ongoing nutritional starvation.

Although previous studies on MTB highlighted a response to nutrient starvation and intramacrophage conditions by up-regulating genes involved in the degradation of lipids or inhibiting lipid synthesis [60,75], both in acid-nitrosative conditions and in macrophage infection, MAP down-regulates the lipid degradation and up-regulates the synthesis of lipids. This is indeed complementary to the up-regulation of genes that participates in the synthesis of LPS, phospholipids and mycolic acids especially in THP-1 infection with concomitant inhibition of genes coding for proteins required for the synthesis of cell wall polysaccharides, especially peptidoglycan. Therefore it can be inferred that, in presence of phagosomal environment, MAP makes use of a kind of primary defense for its own surface that, from the structural point of view, is no longer strictly "rigid" such as found in the acid-nitrosative stress with the strengthening of peptidoglycan which reveals a typical physical-chemical stress, but rather “dynamic and interactive”. This could be explained supposing a sort of “bacterial cell wall spoliation” as a result of the interaction of the infectious agent with the macrophages defence system. MAP would not repair degraded polysaccharides, however restores lipid structures less xenogenic to host cell, since hydrophobicity of lipids makes them less accessible to the immune system than are hydrophophilic molecules such as carbohydrates [76], thus implementing a kind of internal mimicry within intra-macrophage environment by appearing as “self compartment”. This could lead to an incomplete phagosomal acidification following the mycobacterial infection of macrophages [77], thereby avoiding the immune response which would confirm the identification of “cell wall deficient/defective” MAP cells as a way of persistence of the bacterium inside the host as described by several authors [8,78,79].

Finally, within the transcriptome of MAP in macrophage infection, it is worth noting the up- regulation of the gene coding for *hemolysin A* (*tlyA*) while the *hbha* gene is down-regulated. Whereas HBHA protein has been recognized as an important factor which is responsible for the adhesion and invasion in the host cell [80], hemolysin may be considered instead as an evasion factor [81]. In this way, it could be hypothesized that MAP inside macrophage employs a virulence system devoted to escaping from the phagocytic cell, thus limiting invasion. This hypothesis could be consistent with the above-mentioned up-regulation of cell division, thus deducing an increased intracellular proliferation in anticipation of an impending escape from the phagosome, although this should be necessarily taken into account in relation to the temporal stage of MAP infection. However, the concomitant down-regulation of *nuoG*, would reflect the repression of the antiapoptotic effect that bacteria have on the macrophage [63] confirming the hypothesis of evasion and macrophage killing.

## Conclusions

In conclusion, this work showed how MAP's transcriptome, both in the simulation of intraphagosomal acid-nitrosative stress and in macrophage infection, shifts towards an adaptive metabolism for anoxic environment and nutrient starvation, by up-regulating several response factors in order to cope with oxidative stress or intracellular permanence. However, along with the transcriptional similarities between the two types of experiments, especially regarding the energy metabolism, the discovery of significant differences in cell wall metabolism, virulence and antigenical profile between MAP's transcriptomes under acid- nitrosative stress and macrophage infection, makes us understand how the *in vitro* simulation of intracellular stresses and the cell infection act differently in fine regulation of MAP's interactome with the host cell. Therefore, it is clear the importance of the need to construct apart from *in vitro* models, also appropriate *in vivo* models that could reveal further transcriptional differences to identify functional characteristics and particular transcriptional aspects regarding environmental stimuli to which the bacterium has to face, thus identifying genes involved in the molecular pathogenesis of MAP*-*induced diseases.

## Abbreviations

(MAP), Mycobacterium avium subsp. paratuberculosis; (MTB), Mycobacterium tuberculosis; (ADC), Albumin – Dextrose – Catalase; (MicJ), Mycobactin-J; (PMA), Phorbol 12-myristate 13-acetate; (MOI), Multiplicity of infection; (RT), Room temperature; (tot-RNA), Total RNA; (aRNA), Amplified RNA; (DTT), Dithiothreitol; (MeV), Multi Experiment viewer; (KEGG), Kyoto Encyclopedia of Genes and Genomes; (MBGD), Microbial Genome Database; (NCBI-GEO), National Center for Biotechnology Information - Gene Expression Omnibus; (PHAs), Polyhydroxyalkanoic acids; (PTS), Phosphotransferase system; (LPS), Lipopolysaccharide; (MSMEG), Mycobacterium smegmatis.

## Competing interests

The study does not present any conflict of interest for the authors.

## Authors' contributions

Conceived and designed the experiments: AC, VR. Performed the experiments: AC, VR. Analyzed the data: AC, VR. Contributed reagents/materials/analysis tools: AC, VR. Contributed strains/ Instruments tools: LAS, SZ . Wrote the paper: AC, VR. All authors read and approved the final manuscript.

## Authors' information

AC: Young researcher, Department of Biomedical Sciences, Division of Experimental and Clinical Microbiology, University of Sassari, ITALY.

LAS: Full Professor, Department of Biomedical Sciences, Division of Experimental and Clinical Microbiology, University of Sassari, ITALY.

SZ: Full Professor, Department of Biomedical Sciences, Division of Experimental and Clinical Microbiology, University of Sassari, ITALY.

VR: Young Researcher, Experimental Zooprophylactic Institute of Sardinia, Department of Nuoro, ITALY.

## Supplementary Material

Additional file 1 **Additional tables (Tables S1-S4).Table S1.** Genes of *M. avium* subsp*. paratuberculosis* with significantly up-regulated expression levels in the acid-nitrosative stress (≥2 fold change). **Table S2**. Genes of *M. avium* subsp*. paratuberculosis* with significantly down- regulated expression levels in the acid-nitrosative stress (≤2 fold change). **Table S3**. Genes of *M. avium* subsp*. paratuberculosis* with significantly up-regulated expression levels in the infection of THP-1 cells (≥2 fold change). **Table S4**. Genes of *M. avium* subsp*. paratuberculosis* with significantly down-regulated expression levels in the infection of THP-1 cells (≤2 fold change). Click here for file
